# Cryptic transmission and novel introduction of Dengue 1 and 2 genotypes in Colombia

**DOI:** 10.1093/ve/veae068

**Published:** 2024-08-31

**Authors:** David Martínez, Marcela Gómez, Carolina Hernández, Sandra Campo-Palacio, Marina González-Robayo, Marcela Montilla, Norma Pavas-Escobar, Catalina Tovar-Acero, Lillys Geovo-Arias, Esilda Valencia-Urrutia, Nayade Córdoba-Renteria, Marlen Y Carrillo-Hernandez, Julian Ruiz-Saenz, Marlen Martinez-Gutierrez, Alberto Paniz-Mondolfi, Luz H Patiño, Marina Muñoz, Juan David Ramírez

**Affiliations:** Centro de Investigaciones en Microbiología y Biotecnología-UR (CIMBIUR), Facultad de Ciencias Naturales, Universidad del Rosario, Bogotá, Colombia; Centro de Investigaciones en Microbiología y Biotecnología-UR (CIMBIUR), Facultad de Ciencias Naturales, Universidad del Rosario, Bogotá, Colombia; Grupo de Investigación en Ciencias Básicas (NÚCLEO) Facultad de Ciencias e Ingeniería, Universidad de Boyacá, Tunja, Colombia; Centro de Investigaciones en Microbiología y Biotecnología-UR (CIMBIUR), Facultad de Ciencias Naturales, Universidad del Rosario, Bogotá, Colombia; Centro de Tecnología en Salud (CETESA), Innovaseq SAS, Bogotá, Colombia; Molecular Microbiology Laboratory, Department of Pathology, Molecular and Cell-based Medicine, Icahn School of Medicine at Mount Sinai, New York, NY 10029, USA; Laboratorio de Salud Pública, Secretaría de Salud Departamental Meta, Villavicencio, Colombia; Laboratorio de Salud Pública, Secretaría de Salud Departamental Meta, Villavicencio, Colombia; Laboratorio de Salud Pública, Secretaría de Salud Departamental Meta, Villavicencio, Colombia; Universidad Cooperativa de Colombia, Villavicencio, Colombia; Laboratorio de Salud Pública, Secretaría de Salud Departamental Meta, Villavicencio, Colombia; Universidad Cooperativa de Colombia, Villavicencio, Colombia; Grupo de Enfermedades Tropicales y Resistencia Bacteriana, Universidad del Sinú, Montería, Córdoba, Colombia; Secretaria de Salud departamental Chocó-Laboratorio de Salud Pública, Chocó, Colombia; Secretaria de Salud departamental Chocó-Laboratorio de Salud Pública, Chocó, Colombia; Secretaria de Salud departamental Chocó-Laboratorio de Salud Pública, Chocó, Colombia; Grupo de Investigación en Ciencias Animales-GRICA, Universidad Cooperativa de Colombia, Bucaramanga, Colombia; Programa de Estudio y Control de Enfermedades Tropicales-PECET, Universidad de Antioquia, Medellín, Colombia; Grupo de Investigación en Ciencias Animales-GRICA, Universidad Cooperativa de Colombia, Bucaramanga, Colombia; Grupo de Investigación en Ciencias Animales-GRICA, Universidad Cooperativa de Colombia, Bucaramanga, Colombia; Programa de Estudio y Control de Enfermedades Tropicales-PECET, Universidad de Antioquia, Medellín, Colombia; Molecular Microbiology Laboratory, Department of Pathology, Molecular and Cell-based Medicine, Icahn School of Medicine at Mount Sinai, New York, NY 10029, USA; Centro de Investigaciones en Microbiología y Biotecnología-UR (CIMBIUR), Facultad de Ciencias Naturales, Universidad del Rosario, Bogotá, Colombia; Centro de Investigaciones en Microbiología y Biotecnología-UR (CIMBIUR), Facultad de Ciencias Naturales, Universidad del Rosario, Bogotá, Colombia; Molecular Epidemiology Laboratory, Instituto de Biotecnología-UN (IBUN), Universidad Nacional de Colombia, Bogotá, Colombia; Centro de Investigaciones en Microbiología y Biotecnología-UR (CIMBIUR), Facultad de Ciencias Naturales, Universidad del Rosario, Bogotá, Colombia; Molecular Microbiology Laboratory, Department of Pathology, Molecular and Cell-based Medicine, Icahn School of Medicine at Mount Sinai, New York, NY 10029, USA

**Keywords:** dengue virus, genomic epidemiology, phylogenomics, cryptic transmission, cosmopolitan genotype

## Abstract

Dengue fever remains as a public health challenge in Colombia, standing as the most prevalent infectious disease in the country. The cyclic nature of dengue epidemics, occurring approximately every 3 years, is intricately linked to meteorological events like El Niño Southern Oscillation (ENSO). Therefore, the Colombian system faces challenges in genomic surveillance. This study aimed to evaluate local dengue virus (DENV) transmission and genetic diversity in four Colombian departments with heterogeneous incidence patterns (department is first-level territorial units in Colombia). For this study, we processed 266 serum samples to identify DENV. Subsequently, we obtained 118 genome sequences by sequencing DENV genomes from serum samples of 134 patients infected with DENV-1 and DENV-2 serotypes. The predominant serotype was DENV-2 (108/143), with the Asian-American (AA) genotype (91/118) being the most prevalent one. Phylogenetic analysis revealed concurrent circulation of two lineages of both DENV-2 AA and DENV-1 V, suggesting ongoing genetic exchange with sequences from Venezuela and Cuba. The continuous migration of Venezuelan citizens into Colombia can contribute to this exchange, emphasizing the need for strengthened prevention measures in border areas. Notably, the time to most recent common ancestor analysis identified cryptic transmission of DENV-2 AA since approximately 2015, leading to the recent epidemic. This challenges the notion that major outbreaks are solely triggered by recent virus introductions, emphasizing the importance of active genomic surveillance. The study also highlighted the contrasting selection pressures on DENV-1 V and DENV-2 AA, with the latter experiencing positive selection, possibly influencing its transmissibility. The presence of a cosmopolitan genotype in Colombia, previously reported in Brazil and Peru, raises concerns about transmission routes, emphasizing the necessity for thorough DENV evolution studies. Despite limitations, the study underscores genomic epidemiology’s crucial role in early detection and comprehension of DENV genotypes, recommending the use of advanced sequencing techniques as an early warning system to help prevent and control dengue outbreaks in Colombia and worldwide.

## Introduction

Dengue fever, a viral illness transmitted by mosquitoes, has become a global concern, exhibiting both endemic and epidemic patterns (Bhatt et al. 2013). In 2023, it reached a critical juncture, being declared an epidemic year with the highest number of documented cases worldwide, including 2.9 million in the Americas alone during the first half of the year (Informe de situación 2024). The causative agent of this disease is the dengue virus (DENV), primarily transmitted by *Aedes aegypti* and *Aedes albopictus* mosquitoes. DENV is a single-stranded RNA virus with a genome size of ∼10.7 kb, encompassing three structural genes (C, prM, and E) and seven nonstructural genes (NS1, NS2A, NS2B, NS3, NS4A, NS4B, and NS5) surrounded by noncoding regions known as UTR-5' and UTR-3' (Sim and Hibberd 2016, Murugesan and Manoharan 2020). DENV is classified into four serotypes—DENV-1, DENV-2, DENV-3, and DENV-4—based on envelope antigens. They share between 65% and 70% amino acid similarity (Holmes and Twiddy 2003, Katzelnick et al. 2017). Furthermore, each of the four dengue serotypes is subdivided into genotypes. A genotype, in this context, refers to a cluster of DENVs exhibiting no more than a 6% nucleotide divergence (Rico-Hesse 1990). Related to the distribution of these genotypes in the Americas, findings from a systematic review conducted by Ramos-Castañeda et al. in 2017 indicate that all four serotypes have circulated at least once in Latin America’s history (Ramos-Castañeda et al. 2017). However, the prevalent genotypes in the region are DENV-1 genotype V and DENV-2 Asian-American (AA) (Carrillo-Hernandez et al. 2021). Additionally, the recent introduction of the Cosmopolitan DENV-2 genotype has been documented, originating from Asia to Peru and subsequently reported in Brazil and Colombia (Garcia et al. 2022, Amorim et al. 2023, Ciuoderis et al. 2023, Martínez et al. 2024).

While the impact of these circulating genotypes is not fully understood, it is recognized that genetic variations play a pivotal role in the evolutionary dynamics of DENV, particularly in hyperendemic areas (Ko et al. 2020). The genomic analysis has significantly advanced our comprehension of the genomic diversity of DENV. It has unveiled the co-circulation of distinct lineages from the same or different genotypes within specific geographical zones (Cunha et al. 2016). The application of genomic epidemiology in studying DENV among humans has provided insights into the circulation dynamics of lineages within specific geographical areas. Over time, these lineages tend to circulate for a defined period before being replaced by another set of variants—a phenomenon aptly termed “lineage replacement.” In some instances, such replacements have been linked to changes in the virus’s virulence (Zhang et al. 2005). Moreover, the phenomenon of lineage replacement often coincides with shifts in the prevalence of dengue serotypes. This pattern has been observed in extensive studies conducted across various regions worldwide. Notably, countries like Thailand (Southeast Asia), Nepal (South Asia), Brazil, Ecuador, and Colombia (South America) have been key areas where this phenomenon has been identified (Salvo et al. 2019, Brito et al. 2021, Ngwe Tun et al. 2021, Márquez et al. 2023). In these locations, investigations revealed lineages with sequences that come from samples collected at a particular time frame. Crucially, this process of lineage replacement aligns with changes in serotype incidence and, consequently, corresponds to re-emergence and increase in the number of reported dengue cases (Ma et al. 2021).

In Latin America, especially in Brazil, the intricate dynamics of lineage replacement and the circulation of genetic variants of DENV have been thoroughly examined. The research has highlighted multiple introductions of the virus into Brazil, originating from various cross-border pathways with neighboring countries (Dutra et al. 2017, Brito et al. 2021). Interestingly, these newly introduced virus variants also have nationwide transmission with subsequent reporting in different states. Notably, the studies underscore that the emergence of new lineages or genotypes correlates with spikes in dengue cases in those specific regions (Dutra et al. 2017, Salvo et al. 2019, Ma et al. 2021). Conversely, the characterization of DENV genomic variant transmission in Colombia has been relatively limited (Velandia-Romero et al. 2020). However, noteworthy is the recent report regarding the introduction of the Cosmopolitan DENV-2 genotype and the study conducted by Salvo, M. et al., (2019), where the circulation of two lineages of the DENV-1 V genotype was found (Salvo et al. 2019, Ciuoderis et al. 2023, Martínez et al. 2024). In this study, the authors observed higher ratios of nonsynonymous to synonymous mutations among the nonstructural genes compared to the structural genes. These findings suggest that positive selection may be a driving force in the evolution of DENV within local communities. This underscores the role of genomic epidemiology in comprehending DENV evolution. Consequently, it highlights the significance of ongoing efforts to understand the interplay between epidemiological patterns and viral genetics—genomic epidemiology—emphasizing its role as a crucial tool for public health surveillance. This approach is pivotal for crafting more effective and locally designed prevention and control programs.

In Colombia, according to the 2023 annual report from the National Institute of Health (Instituto Nacional de Salud) up to epidemiological Week 52, there have been 131 784 probable cases of dengue. The departments reporting the highest cases are Meta, Valle del Cauca, and Tolima. Consequently, our study initially aimed to identify the most prevalent dengue serotypes in Colombia (Carrillo-Hernandez et al. 2021) in four departments: Chocó, Meta, Monteria, and Norte de Santander. Subsequently, we sequenced the complete genome of DENV from positive samples to identify the circulating genotypes. To achieve this, we implemented and standardized a methodology using Oxford Nanopore Technologies (ONT). Finally, through comprehensive genomic analyses, we were able to describe the potential transmission routes and genetic diversity of each lineage within the identified genotypes.

## Materials and methods

### Ethics statement

The Technical Research Committee and the Research Ethics Council at the University of Rosario in Bogotá, Colombia, approved the protocol implemented in this study DVO005 2438-CV1781: “Genomic Characterization of the Dengue Virus in patients and vectors from different biogeographic regions of Colombia.”

### Sample collection

**Figure 1.**
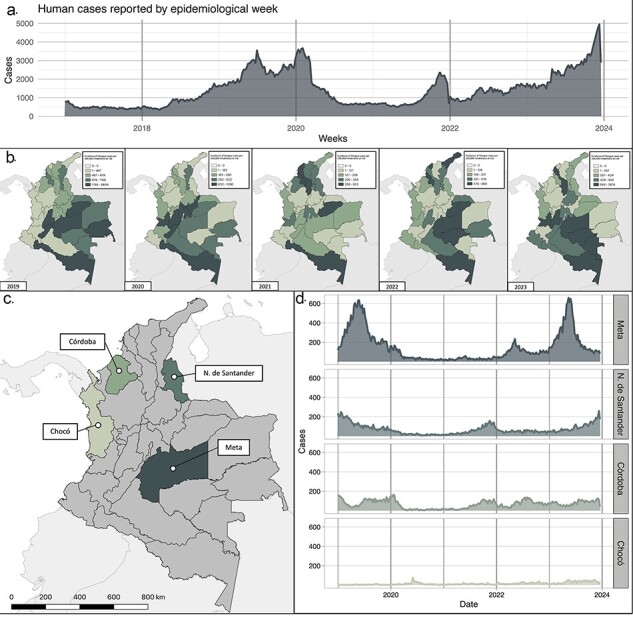
Dengue incidence in Colombia from 2017 to 2023 and study sites. (a). Weekly reported dengue cases in Colombia from Week 1 of 2017 to Week 52 of 2023. (b) Map of Colombia depicting dengue incidence per 100 000 at-risk inhabitants by department from 2019 to 2023. (c) Map of Colombia highlighting the four study departments: Meta, Norte de Santander, Córdoba, and Chocó. (d) Weekly reported dengue cases at study sites from Week 1 of 2019 to Week 52 of 2023. Colors indicate study sites and correspond to the map in (c). The data used for these figures were obtained from Colombia’s Public Health Surveillance System (Sivigila).

A total of 261 serum samples of patients with suspected dengue were included in this study from three departments in Colombia, collected between the years 2022 and 2023: Chocó (*n* = 24), Meta (*n* = 212), and Córdoba (*n* = 25) (Fig. 1). These serum samples were obtained and provided by public and/or private entities responsible for their diagnosis (Departmental Public Health Laboratory of Chocó, Departmental Public Health Laboratory of Meta, and Biomedical Research Laboratory of the University of Sinú in the Department of Córdoba). Additionally, five sera from the Department of Norte de Santander were included, collected in the years 2015 and 2019 (Animal Sciences Research Group-Grupo de Investigaciones en Ciencias Animales, Cooperative University of Colombia, Bucaramanga). This was done to incorporate them into the phylogenetic analyses described later. The entities provided 200 µl of serum in vials stored at −80°C. Finally, the samples were transported to the microbiology laboratory at the University of Rosario in Bogotá, Colombia, for processing and molecular analysis.

### Sample processing, RNA extraction, and multiplex polymerase chain reaction for DENV serotype identification

RNA was extracted from 200 µl of serum using the Quick-RNA Viral-ZYMO kit (Zymo, ref 1035), following the manufacturer’s recommended instructions. After obtaining the genetic material, its concentration and quality were evaluated using the Nanodrop-2000 spectrophotometer (Thermo Fisher Scientific, Waltham, MA, USA), and it was stored at −80°C. Subsequently, reverse transcription polymerase chain reaction (RT-PCR) was carried out from the genetic material to generate complementary DNA (cDNA) using the LunaScript RT SuperMix Reverse Transcriptase Kit (New England Biolabs #E3010). The cDNA was stored at −30°C.

The detection of DENV serotypes (DENV 1–4) was performed using a multiplex PCR, employing primers for the C-prM gene region as previously reported (Table 1; Chien et al. 2006) (Chien et al. 2006). The GoTaq®Green PCR Master Mix (2×) enzyme (Promega, no. M7123) was used with 10 µmol of each primer (MD1, rTS1, rTS2, rTS3, and rTS4) and 0.8 µl of cDNA. The thermal profile consisted of an initial denaturation cycle at 95°C for 5 min, followed by 35 cycles of 95°C for 30 s, 57°C for 45 s, and 72°C for 33 s, finally a single extension cycle at 72°C for 10 min. Amplified fragments were visualized using 2% agarose gel electrophoresis in 1× TBE buffer with 1 μl of SYBR® Safe (Invitrogen®, Carlsbad, CA, USA) as an intercalating agent. The gel was then placed under UV light to observe the amplification band for each serotype: 208 base pairs (bp) for DENV-1, 119 bp for DENV-2, 288 bp for DENV-3, and 260 bp for DENV-4. Positive controls for DENV (supernatant from cells individually infected with DENV-1, DENV-2, DENV-3, and DENV-4 serotypes) were provided by the Universidad Cooperativa de Colombia. Finally, the confirmation of positive samples was conducted in duplicate.

**Table 1.** List of primers used in PCR for the detection of DENV, serotypes DENV-1–4 (Chien et al. 2006).

DENV detection					
Virus	Primer/probe	Sequence	Genomic position	Amplicon size (bp)	Genomic region amplified
DENV-1	DENV1—F	mD1-TCAATATGCTGAAACGCGHGAGAAACCG	134–322	208	C-prM
	DENV1—R	rTS1-CCCGTAACACTTTGATCGCT			
DENV-2	DENV2—F	mD1-TCAATATGCTGAAACGCGHGAGAAACCG	134–232	119	
	DENV2—R	rTS2-CGCCACAAGGGCCATGAACAGTTT			
DENV-3	DENV3—F	mD1-TCAATATGCTGAAACGCGHGAGAAACCG	134–400	288	
	DENV3—R	rTS3-TAACATCATCATGAGACAGAGC			
DENV-4	DENV4—F	mD1-TCAATATGCTGAAACGCGHGAGAAACCG	134–374	260	
	DENV4—R	rTS4-TTCTCCCGTTCAGGATGTTC			

### Genome sequencing of DENV using ONT

The cDNA obtained in the previous step from DENV-1- and DENV-2-positive samples was used as a template for whole-genome amplification by a multiplex PCR, employing the DENV sequencing scheme previously reported by Stubbs et al. (2020). Two multiplex PCR reactions were conducted per sample, with primers separated into two groups (Pool-1 and Pool-2) to prevent interference between overlapping amplicons (Supplementary Table S1). PCR reactions were performed in a final volume of 12.5 µl, containing 1.25 µl of cDNA reaction mixture, 6.25 µl of Q5 polymerase (NEB), and a variable volume of either primer Pool 1 or 2 (10 μM) to a final concentration of 0.015 μM per primer (e.g. 1.5 μl for DENV-1, containing 40 primers per group). RNase-free water was added to achieve the final volume. The thermal profile for the two-step PCR amplification is as follows: an initial denaturation cycle at 98°C for 30 s, followed by 40 cycles of 98°C for 15 s and 65°C for 5 min. After amplification, the concentrations of the two reaction groups were quantified using the Qubit dsDNA HS kit with a Qubit 3.0 fluorometer (ThermoFisher Scientific Corporation, Waltham, MA, USA). This was done to consolidate the two reactions per sample and obtain a final concentration of 10 ng/µl for use in sequencing.

The amplified products were sequenced using ONT, starting with barcode ligation (assigning one barcode per sample) using the ONT Barcode Kit (EXP-NBD196). Subsequently, the library was formed by pooling equal volumes of amplicons (already with their linked barcode), followed by adapter ligation using the ONT ligation sequencing kit (SQK.LSK109). The formed library was sequenced on the ONT MinION using R.9.4 flow cells and MinKnow V.3.1.4 software. Bioinformatic analysis was performed using raw Fast5 files, which underwent Super Accuracy basecalling (SUP) (*Q* > 10) to obtain Fastq files. Subsequently, demultiplexing was conducted using the Guppy V3.1.5 tool (Wick et al. 2019).

### Genome assembly and genotyping

Viral genome assembly was performed using Fastq files through mapping assembly with the Minimap2 tool (V.2.26) (Li and Birol 2018), using NC_001477.1 (for DENV-1) and NC_001474.2 (for DENV-2) as reference genomes. Consensus sequences and variant calling were obtained using the Nanopolish tool (V.0.14) (Hu et al. 2021), which cross-checks the assembly with the original sequencing data (Fast5). Additionally, a minimum depth of at least 20 reads per site was established. In cases where this parameter was not met, “*N*” bases were assigned at the corresponding positions in the consensus sequence. The consensus sequences were used for genotype identification in the online Dengue Virus Typing Tool (https://www.genomedetective.com/app/typingtool/dengue/). Genotype assignment is based on the location of genomes within the phylogenetic reconstruction, satisfying the following conditions: (i) it must present a monophyletic grouping with sequences of the assigned genotype and (ii) it must have a bootstrap support >70% (Fonseca et al. 2019). It is important to mention the new nomenclature that is presented to homogenize the classification of DENV, where the genotype classification is maintained, but two more levels are added within the genotypes, major and minor lineages (Hill et al. 2024).

### Selection of DENV genomic data and alignment

The comparison dataset was constructed from complete genome sequences available in the NCBI DENV database (DENV-1 and DENV-2) (https://www.ncbi.nlm.nih.gov/genomes/VirusVariation/Database/nph-select.cgi). Sequences were downloaded for the three genotypes commonly circulating in the Americas: DENV-1 genotype V (*n* = 715), DENV-2 genotype AA (*n* = 933), and DENV-2 genotype Cosmopolitan (*n* = 701). For subsequent analyses, only genomes with known collection dates and locations were included (Supplementary Table S2). These downloaded sequences were combined with those obtained in this project, and a multiple alignment was performed using MAFFT V.7 with the FFT-NS-2 algorithm and default parameters (Katoh et al. 2005). Subsequently, UTR regions were removed using the Unipro UGENE program v.33.0 (Okonechnikov et al. 2012). Maximum likelihood (ML) analysis was conducted from these alignments using IQtree software (Nguyen et al. 2015) with the nucleotide substitution model that best fit the data and 1000 bootstrap replicates for node support.

### Discrete phylogeographic analysis

To unravel the evolutionary and dispersal history of DENV-1 genotype V, DENV-2 genotype AA, and DENV-2 genotype Cosmopolitan in Latin America and primarily in Colombia, a Bayesian phylogenetic inference was conducted using BEAST v.1.10.4 (Suchard et al. 2018). Based on the selection of molecular clock models, it was identified that the relaxed clock model best suited the project’s data. Accordingly, a coalescent tree—Bayesian Skyline—was built with a GTR + Γ4 nucleotide substitution model and a relaxed molecular clock. Geographical data of sequence origins were organized by continent, and those from South America were further classified by country. Ancestral origins of genotypes circulating in the Americas were inferred using the reversible discrete phylogeography model. Implementing the BEAGLE v.3.1 (Ayres et al. 2019) tool to accelerate analysis, Markov Chain Monte Carlo (MCMC) chains were sampled for 100 million iterations and parameter convergence (ESS > 200) was determined using Tracer v.1.7.1 (Rambaut et al. 2018). Subsequently, a 10% burn-in was discarded and maximum credibility trees were summarized using TreeAnnotator v1.10.4 (Suchard et al. 2018). The results were visualized using the online tool iToL and plotted on a geographical map using SpreaD3 v.0.9.7.1 (Bielejec et al. 2016).

### Continuous phylogeographic analysis

The dispersal history of DENV genotypes circulating in Colombia was inferred using a continuous phylogeographic method implemented in BEAST v.1.10.4 (Suchard et al. 2018). This analysis focused on the three monophyletic clades of DENV-1 genotype V, DENV-2 genotype AA, and DENV-2 genotype Cosmopolitan from the genomes sequenced in this study. The previously described evolutionary model was employed, utilizing the highest posterior density (95%) from these analyses as normal prior distributions to establish the TMRCA of the clades. Considering the geographical coordinates associated with the collection location of each sequence, the Cauchy relaxed random walk diffusion model was used to infer the coordinates associated with the ancestors of the sequences (internal nodes). Implementing the BEAGLE v.3.1 (Ayres et al. 2019) tool to accelerate the analysis, MCMC chains were sampled for 150 million iterations and the parameter convergence (ESS > 200) was determined using Tracer v.1.7.1 (Rambaut et al. 2018). Subsequently, a 10% burn-in was discarded and maximum credibility trees were summarized using TreeAnnotator v1.10.4 (Suchard et al. 2018). Finally, the results were plotted in geographical space using SpreaD3 v.0.9.7.1.

### Estimation of dN/dS and single-nucleotide polymorphism analysis

Initially, the ratio of nonsynonymous to synonymous changes (d*N*/d*S*) was calculated for each pair of sequences and for each gene, using the ML method implemented in CodeML within the PAML tool v.4.10.7 (Gao et al. 2019) with the following settings: runmode = −2 and CodonFreq = 2. Subsequently, the average d*N*/d*S* was calculated by taking the ratio of the average d*N* to the average d*S*. A d*N*/d*S* ratio < 1 indicates the possibility of purifying selection acting to eliminate this new variant. A d*N*/d*S* ratio of ≈1 suggests neutral evolution or alternatively that genetic drift plays a predominant role in the variation of this section. A d*N*/d*S* ratio > 1 indicates that positive selection is acting to favor new polymorphisms. Additionally, a single-nucleotide polymorphism (SNP) analysis was performed directly from the alignment of whole-genome sequences using the SNP site script (available at https://github.com/sanger-pathogens/snp-sites). This analysis was visualized using a heatmap graph through the online heatmapper tool (available at http://heatmapper.ca).

## Results

### Dengue incidence in Colombia and sampling characteristics

The number of reported dengue cases fluctuates annually, but the overall trend in the country since 2008 reveals epidemic cycles with increased cases every 3 years. In 2019, 124 989 cases were reported (Fig. 1a), marking the highest annual infection rate since the initiation of DENV transmission in the country. However, at the onset of 2020, a decrease in reported cases occurred, primarily due to undersampling during the Coronavirus Disease (COVID-19) pandemic health emergency. The years 2020 and 2021 are considered interepidemic periods, witnessing a reduction in cases, with fewer than 1000 cases reported per epidemiological week and an incidence ranging between 172.3 and 295.2 cases per 100 000 inhabitants at risk (Fig. 1a and b). In 2022, there is a resurgence in cases that persist into 2023, reporting 131 784 cases and an incidence of up to 368.6, resembling data seen in 2019, the preceding epidemic year (Fig. 1a and b).

Our study focused on four specific departments: Meta, Norte de Santander, Córdoba, and Chocó (Fig. 1c). These departments are situated in diverse bioregions across the country, each displaying distinct patterns of dengue incidence: Meta (∼1555.3), Norte de Santander (∼366.5), Córdoba (∼220.6), and Chocó (∼135) (Fig. 1b). Historically, Meta has consistently ranked among the top five most affected departments in the country, reporting a substantial number of DENV cases annually. In 2023, Meta contributed 10.2% of the total reported cases in the country, with a count of 12 669 cases (Fig. 1b and d). In contrast, Norte de Santander and Córdoba reported fewer than 300 cases per epidemiological week (Fig. 1b and d). Finally, Chocó, over time, has experienced relatively lower DENV incidence, consistently reporting less than 100 cases per epidemiological week (Figure 1b and d).

### DENV serotype identification and genotyping

A total of 266 samples underwent PCR for serotype identification, resulting in 143 samples testing positive for DENV, with DENV-2 being the most prevalent serotype (108/143) (Table 2). DENV-2 was identified in all four departments, while DENV-1 was found in three departments (Meta, Córdoba, and Norte de Santander). Additionally, only nine positive samples were identified for DENV-3 in the Meta department. Whole-genome sequencing of DENV was carried out for DENV-1- and DENV-2-positive samples (134/143), the most prevalent serotypes in Colombia.

**Table 2.** Number of samples processed, serotyping, and genomes obtained by each department.

		PCR DENV		
Departments	Samples (%)	Serotype	Positives	Genomes
Chocó				
	24 (9.02)	DENV-2	3	1
Meta				
	212 (79.7)	DENV-1	18	17
		DENV-2	99	86
		DENV-3	9	**–**
Córdoba				
	25 (9.4)	DENV-1	4	4
		DENV-2	5	5
Norte de Santander				
	5 (1.88)	DENV-1	4	4
		DENV-2	1	1
Total	266		143	118

Sequencing efforts yielded 118 DENV sequences with at least 50% genome coverage (Table 2). Of these, 103 sequences were from individuals in various cities within the Meta department, nine from Córdoba, five from Norte de Santander, and one from Chocó. Most of the sequences were obtained during the recent epidemic period of 2022–3 (113/118). Genotype assignment analysis revealed 25 sequences of DENV-1 genotype V (Supplementary Fig S1), 91 sequences of DENV-2 genotype AA (Supplementary Fig S2), and 2 sequences of DENV-2 Cosmopolitan genotype (Supplementary Fig S3). The DENV-2 genotype AA was predominantly identified in the Meta department (86/102; Table 2), with the DENV-2 Cosmopolitan genotype exclusively found in this same department.

### Phylogenetic analysis of DENV-1 genotype V and its spatial dispersion

**Figure 2.**
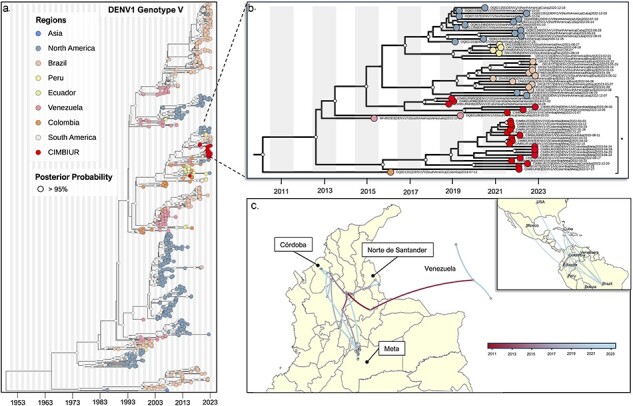
Emergence and cryptic transmission of DENV-1 genotype V in Colombia. (a) Time-resolved phylogeny of DENV-1 genotype V (*n* = 715). Point colors represent the origin of each sequence, with sequences from this study (*n* = 25) highlighted in red. (b) Clade containing sequences from this study. (c) Continuous phylogeographic analysis showing local spread of DENV-1 genotype V in Colombia during 2011–23 (gradient bar from red to blue).

The Maximum Clade Credibility (MCC) tree, constructed using BEAST with complete genome sequences of DENV-1 genotype V, reveals that the sequences from this study were grouped within the clade with the larger proportion of Colombian sequences, suggesting the circulation of this clade in Colombia since approximately 1996 [95% Bayesian credible interval (BCI) 1995.11–1997.03; Fig. 2a]. Notably, the sequences from this study segregate into two lineages, with one exhibiting a close association with sequences from Venezuela and Cuba (Fig. 2b). This indicates two instances of transborder viral exchange, as supported by the phylodynamic analysis: (i) an introduction from Colombia to Venezuela, estimated to have occurred in 2014 (95% BCI 2012.05–2015.02; Fig. 2b and c), and (ii) an introduction from Cuba to Colombia, estimated to have taken place in 2015 (95% BCI 2014.22–2017.89; Fig. 2b and c). Examining internal dispersion within Colombia, the geographical plot illustrates continuous and active transmission among the sampled departments, highlighting the crucial role of intermediary departments such as Antioquia, Santander, and Bolívar in facilitating this transmission (Fig. 2c). It is important to highlight that for these departments, there are no sequences available in NCBI despite the estimated presence of disease in certain regions in the country.

### Phylogenetic analysis of DENV-2 genotype AA and spatial dispersion

**Figure 3.**
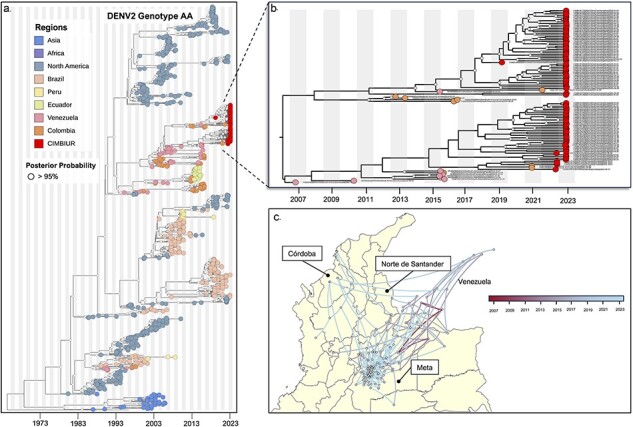
Emergence and cryptic transmission of DENV-2 genotype AA in Colombia. (a) Time-resolved phylogeny of DENV-2 genotype AA (*n* = 933). Point colors represent the origin of each sequence, with sequences from this study (*n* = 91) highlighted in red. (b) Clade containing most sequences from this study. (c) Continuous phylogeographic analysis showing local spread of DENV-2 genotype AA in Colombia during 2007–23 (gradient bar from red to blue).

The MCC tree constructed for the complete genome sequences of DENV-2 genotype AA indicates that sequences from this study form a clade alongside other Colombian sequences. The estimated circulation of this clade in Colombia dates to around 2000 (95% BCI 1998.48-2002.85; Fig. 3a). Specifically, the sequences from this study segregate into two lineages, both of which are linked to sequences obtained from Venezuela (Fig. 3b). This suggests multiple transborder viral exchanges with Venezuela, including (i) three introductions to Colombia from Venezuela estimated to have occurred in 2008 (95% BCI 2007.54–2010.83; Fig. 3b), 2012 (95% BCI 2011.66–2014.6; Fig. 3b), and 2015 (95% BCI 2014.54–2017.92; Fig. 3b) and (ii) three introductions to Venezuela from Colombia are dated to the years 2009 (95% BCI 2007.83–2011.88; Fig. 3b), 2016 (95% BCI 2014.27–2017.63; Fig. 3b), and 2017 (95% BCI 2016.46–2018.37; Fig. 3b). The phylogeographic analysis further indicates continuous and active transmission among Colombian departments (Antioquia, Bolívar, Santander, Casanare, Arauca, and Vichada) and with Venezuela (Apure, Barinas, Carabobo, and Aragua), evidenced by various identified introductions (Fig. 3c).

### Phylogenetic analysis of DENV-2 Cosmopolitan Genotype and spatial dispersion

**Figure 4.**
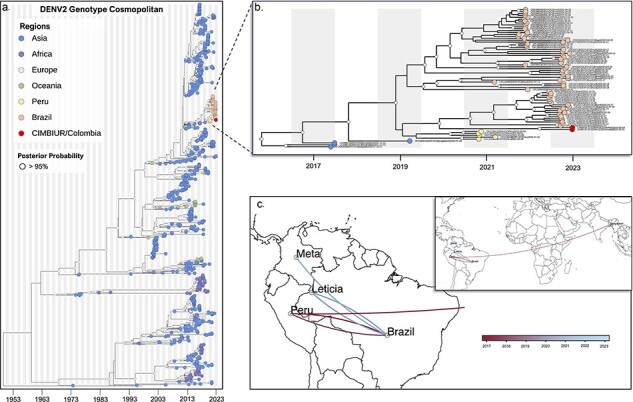
Emergence and cryptic transmission of DENV-2 Cosmopolitan genotype in Colombia. (a) Time-resolved phylogeny of DENV-2 Cosmopolitan Genotype (*n* = 701). Point colors represent the origin of each sequence, with sequences from this study (*n* = 2) highlighted in red. (b) Clade containing most sequences from this study. (c) Continuous phylogeographic analysis showing local spread of DENV-2 Cosmopolitan Genotype in Colombia during 2017–23 (Gradient bar from red to blue).

The MCC tree, generated using BEAST for complete genome sequences of Cosmopolitan DENV-2 genotype, indicates that the two sequences from this study are situated within a clade containing sequences from the Americas, with the estimated date of circulating in the region since 2019 (95% BCI 2019.69–2020.6; Fig. 4a). Specifically, the sequences from this study are closely related to those from Brazil, sampled between late 2022 and early 2023. According to the TMRCA, the introduction event from Brazil to Colombia occurred in February 2020 (95% BCI 2020.88–2021.27; Fig. 4b). Additionally, the phylogeographic analysis suggests a potential introduction from Tabatinga, Brazil, with subsequent northward spread in Colombia (Fig. 4c).

### Diversity and SNP analysis

**Figure 5.**
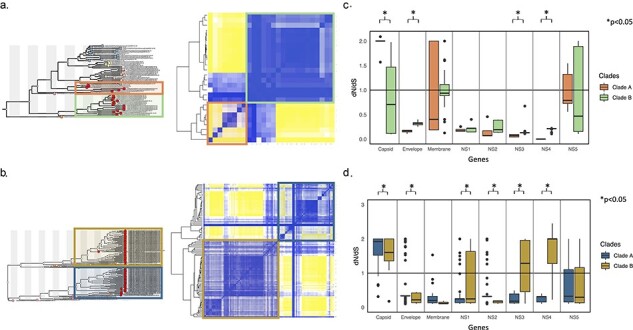
Genetic diversity of DENV-1 genotype V and DENV-2 genotype AA. (a) Identified clades of DENV-1 genotype V and corresponding SNP analysis. (b) Calculation of the ratio of nonsynonymous to synonymous substitutions (d*N*/d*S*) for each gene between Clades A and B of DENV-1 genotype V. (c) Identified clades of DENV-2 genotype AA and corresponding SNP analysis. (d) Calculation of the ratio of nonsynonymous to synonymous substitutions (d*N*/d*S*) for each gene between Clades A and B of DENV-2 genotype AA.

The ML phylogenetic analysis, based on the complete genomes of DENV-1 genotype V and DENV-2 genotype AA, reveals a distinct separation into two clades within each genotype (Fig. 5a and b). The computation of the SNP distance matrix between genomes within each clade confirms their relatedness, indicating specific SNP patterns for each clade (Fig. 5a and b). To delve into the selection pressures within each gene and between clades of each serotype, nucleotide diversity was assessed by calculating the ratio of nonsynonymous to synonymous substitutions (d*N*/d*S*). This analysis demonstrates a tendency toward lower dN/dS ratios in Clades A and B corresponding to DENV-1 genotype V (Fig. 5c), suggesting that these genes may be subject to purifying selection. Conversely, in DENV-2 genotype AA, Clade B exhibits a trend toward higher ratios in the nonstructural genes NS1, NS3, and NS4 (Fig. 5d), indicating a potential influence of positive selection in these genes. Additionally, a significant difference in d*N*/d*S* values between Clades A and B is observed in the NS1, NS3, and NS4 genes of DENV-2 genotype AA (NS1: *P* = 0.009129, NS3: *P* = 5.574 × 10−^13^, NS4: *P* < 2.2e × 10^−16^).

## Discussion

Dengue currently stands as the infectious disease with the highest incidence in Colombia, a circumstance driven by factors like climate change, ever increasing trends in deforestation, the simultaneous circulation of all four serotypes, and the prevalence of their primary vectors across most of the territory (Boletín Epidemiológico Semanal 2023). The annual case numbers exhibit cyclical patterns, presenting epidemics roughly every 3 years (Fig. 1a). This behavior aligns with periods of alternating dominance among the four serotypes, shifting every 3–5 years. Previous research has linked these epidemic cycles to meteorological events such as El Niño and La Niña, directly affecting vector life cycles due to the availability of microenvironments conducive to their development (Muñoz et al. 2021). While similar patterns are observed in various countries across the Americas, the Colombian system faces challenges in genomic surveillance of the DENV. In contrast, Brazil has conducted more detailed studies on this phenomenon. In Brazil, during the recent epidemic spanning 2022 and 2023, a notable shift in serotype prevalence from DENV-2 to DENV-1 was observed (Verma et al. 2023). Subsequent investigations revealed that this change is strongly influenced by factors like the introduction of new genetic variants within circulating genotypes and the simultaneous circulation of different lineages (Brito et al. 2021). Nevertheless, the virulence characteristics of these newly introduced lineages, which may contribute to the displacement of existing lineages, remain poorly understood (Thongsripong et al. 2023). This knowledge gap stems from limited studies on the virulence of variants or lineages, leaving the implications of their mutations unclear. Therefore, urgent attention is required for genomic surveillance and epidemiological studies to uncover crucial information about DENV transmission patterns (Ma et al. 2021).

Therefore, this study aimed to evaluate local DENV transmission and the genetic diversity of circulating genotypes in four departments of Colombia with varying incidence and biogeographic patterns, (Fig. 1b and c). Initially, we identified the serotypes in the provided samples, revealing a higher prevalence of DENV-2 circulation (95/120), aligning with the current situation in other regional countries (Lessa et al. 2023). The recent upswing in DENV-2 cases affirms findings from previous studies, suggesting that DENV serotypes can persist cryptically during periods of low transmission (de Bruycker-nogueira et al. 2016, Dutra et al. 2017). We specifically focused on identifying the circulating genotypes of the two identified serotypes, with DENV-2 genotype AA being the predominant genotype (77.5%; 93/120), followed by DENV-1 genotype V (20.8%; 25/120) (Figs. 2a and 3a). This observed pattern has been previously documented in Africa, where the emergence of DENV-2 resulted in a subsequent increase in cases compared to DENV-1. Moreover, phylogenetic analysis suggests that the emergence of DENV-2 may be linked to the introduction of a new lineage of this serotype (Fourié et al. 2021).

Previously noted in Guangzhou, China, the introduction of a new variant in a defined region can lead to a significant surge in reported cases in the period following its introduction (Ma et al. 2021, Thongsripong et al. 2023). This phenomenon may be attributed to the introduced variant possessing virulence characteristics that enhance its fitness, resulting in the displacement of other circulating variants (Ngwe Tun et al. 2021). However, these virulence characteristics are poorly understood, and the direct consequences of this phenomenon remain unknown. Hence, it underscores the importance of identifying the lineages of circulating genotypes and characterizing transmission dynamics to shed light on possible introductions facilitating the establishment of new variant circulation in the country.

From our phylogenetic analysis, it is evident that two lineages of both DENV-2 genotype AA and DENV-1 genotype V are concurrently circulating in the territory. These sequences show associations with sequences from Venezuela and, in the case of DENV-1 genotype V, with sequences from Cuba (Figs. 2b and 3b). This suggests an ongoing exchange of genetic variants through various introductions in recent years (Figs 2b, c and 3b, c). The continuous migration of Venezuelan citizens into Colombian territory has been previously identified as a significant factor not only in the introduction and transmission of DENV but also in the spread of other infectious diseases (Carrillo-Hernandez et al. 2021). This underscores the need to strengthen prevention and control measures in border areas. Moreover, Colombia has been recognized for its pivotal role in cross-border transmission to other regions in the Americas. For example, a study conducted by Márquez S. et al., 2023, in Ecuador, revealed that virus sequences originating from Colombia are ancestral and represent a potential source of variant introduction to Ecuador (Márquez et al. 2023). This pattern can be linked to migration from Venezuela, as migrants typically pass through Colombia for approximately 5 days before reaching the northern region of Ecuador (Maljkovic Berry et al. 2020, IDEHPUCP 2023). Additionally, it is crucial to highlight this issue for the future, also given the ongoing massive migration involving movement from Colombia to Panama and the USA through the Darien region. Therefore, gaining a comprehensive understanding of variant circulation and transmission patterns will enable the formulation of future control strategies, facilitating interventions to prevent both local and cross-border DENV transmission. From an ecological perspective, this is particularly significant, as the establishment of migration routes through specific ecosystems may heighten exposure to vector and reservoir species, thereby narrowing the interface for exposure and increasing the risk of transmission. This becomes even more pertinent given Colombia’s geographical location as crossroads for multiple migratory routes connecting the highly biodiverse areas of the Mesoamerican corridor to the north and the Andean corridor to the south.

Our study suggests that human migration is the main way DENV variants are introduced into Colombia from Venezuela. However, other ecological factors might also play a role. One such factor is the distribution of the mosquito *A. albopictus*. This mosquito thrives in areas near human settlements (periurban areas) and feeds on different animals, including rodents (Muridae). This behavior allows infected mosquitoes or hosts to cross borders between countries, potentially introducing new DENV variants. However, testing this hypothesis is challenging because it involves several factors, including potential changes in mosquito or host behavior. Therefore, we focused on human migration. We found that migration routes often pass through ecosystems with a high risk of exposure to DENV-carrying mosquitoes and animals.

On the other hand, the TMRCA analysis revealed the circulation of two lineages of DENV-2 genotype AA since approximately 2015 (Fig. 2b). This suggests a potential cryptic transmission lasting 7–8 years, leading to the recent epidemic period that resulted in outbreaks in the sampled departments. A similar scenario was observed during the 2019 outbreak in the southeast and northeast regions of Brazil, where lineages circulating from 5 to 10 years prior re-emerged to cause the outbreak (Dutra et al. 2017). Consequently, the authors suggest that major dengue outbreaks are not always triggered by recent virus introductions. Instead, circulating lineages undergo phases of broad regional dispersion before consolidating in urban areas, contributing to the occurrence of local outbreaks. This highlights the crucial role of active genomic surveillance in the real-time identification of circulating variants in countries. Such a proactive approach is essential for devising preventive control strategies against the cryptic circulation of DENV variants and potential future outbreaks. Alternatively, waning population immunity preceding genotype emergence may contribute to increased DENV activity. We posit that, specifically in the context of genotypes, virus–host interactions warrant investigation alongside the immune response. Prior research has established that these interactions can enhance viral replication and even exacerbate host symptoms. Consequently, the introduction of a novel variant harboring unknown mutations could significantly impact virus–host interactions. Therefore, to comprehensively evaluate this hypothesis, concurrent implementation of immune surveillance alongside genomic surveillance is necessary.

As previously mentioned, due to the limited studies on this topic, the characteristics of virulence and their direct impact on lineage displacement, and consequently, the increase in the number of cases, remain unknown. However, as a preliminary approach to understanding why such behaviors occur, it is essential to explore selection pressures and identify their patterns in each of the virus genes (Li et al. 2022). Therefore, we assessed the selection pressures present in each of the genes of the identified clades by implementing the d*N*/d*S* ratio. The values obtained for DENV-1 genotype V (d*N*/d*S* < 1; Fig. 5a and c) suggest that this genotype is possibly under purifying selection. This implies that the purifying selection pressure prevents mutations from becoming fixed, and if these mutations are related to virulent characteristics, their fitness decreases, impacting their transmission capacity (Salvo et al. 2019). This behavior aligns with the overall pattern of serotypes in our country, where a decrease in the prevalence of DENV-1 is evident, accompanied by an increase in the prevalence of DENV-2.

On the other hand, our analysis revealed that DENV-2 genotype AA (with d*N*/d*S* > 1; Fig. 5b and d), particularly in Clade B, is likely experiencing positive selection pressure. This suggests that the ongoing selection pressure favors the fixation of new mutations over time. As mentioned earlier, if these mutations are associated with virulence, their fitness increases, enhancing their transmission capacity (Li et al. 2022). Notably, the NS1, NS3, and NS4 genes exhibit a significant difference in d*N*/d*S* values between each clade (NS1: *P* = .009129, NS3: *P* = 5.574 × 10^−13^, NS4: *P* < 2.2 × 10^−16^), with Clade B displaying notably high values. This pattern aligns with the biological functions of these genes (NS1, NS3, and NS4) in the virus’s replication and transmission. NS1 and NS3, being involved in genetic material replication, and the antibodies against NS1 have demonstrated protection against subsequent infection by a heterologous serotype (Pollett et al. 2018). Therefore, we can infer that the selection pressure on these genes is likely linked to the increased transmissibility of the variant and, consequently, the effectiveness of its infection.

The latter findings gain further relevance from an ecological and epidemiological perspective, considering that flaviviruses are prone to alternate between different hosts (both vertebrates and invertebrates), thus subjecting them to diverse selective pressures and evolutionary trajectories. Our results clearly demonstrate such divergence of trajectories, with DENV-1 genotype V exhibiting a d*N*/d*S* ratio < 1 and DENV-2 genotype AA showing a d*N*/d*S* ratio > 1. These results not only imply that DENV-1 genotype V is undergoing purifying selection while DENV-2 genotype AA is experiencing positive selection but also prompt us to consider the origins and overlap of these divergent viral genotypes in a multifactorial ecological context. Studies have indicated that hosts may play a determinant role in driving purifying selection in both mosquitoes and tick-borne flaviviruses (Grubaugh et al. 2016a, 2016b), while episodic positive selection influences the evolution of mosquito-borne flaviviruses (Pontremoli et al. 2021).

When comparing the d*N*/d*S* ratio at a single protein level between DENV-1 genotype V and DENV-2 genotype AA, notable differences were observed in both structural [capsid protein (C), envelope protein (E)] and nonstructural proteins (NS1, NS2A, NS2B, NS3, NS4A, NS4B, and NS5). Specifically, for DENV-1 genotype V, a lower d*N*/d*S* ratio was observed in C, E, NS3, and NS4, while for DENV-2 genotype AA, a higher d*N*/d*S* ratio was observed individually in C, E, NS1, NS2, NS3, and NS4 (Fig. 5). This greater d*N*/d*S* ratio suggests ongoing positive selection for DENV-2 genotype AA. Our findings align with those of Pontremoli et al., who noted that positively selected sites in tick- and mosquito-borne flaviviruses are scattered throughout the polypeptide region in all proteins, except for the membrane (M) protein in mosquito-borne viruses (Pontremoli et al. 2021). They also observed a higher frequency of selected sites in nonstructural proteins than in structural proteins, consistent with our observations (Pontremoli et al. 2021). This is significant as instances of positive selection at specific sites have been associated with higher transmission rates in mosquito-borne viruses (Tsetsarkin et al. 2007, 2014, May et al. 2011). Further in-depth studies are warranted to characterize the driving forces shaping the evolutionary landscape of all four dengue serotypes in Colombia, considering diverse host ranges, vectors, environments, and inter- and intrahost evolution.

One of our key findings was the detection of the DENV-2 Cosmopolitan genotype circulating, a variant that has recently expanded its presence in both Africa and the Americas (Yenamandra et al. 2021). This widespread distribution has led to significant diversity within the genotype, reflecting the evolutionary forces influencing its transmission. Notably, an outbreak linked to the cosmopolitan genotype occurred in Peru’s Madre de Dios province in 2019, aligning with its recent emergence in Africa (Garcia et al. 2022). In 2021, additional reports surfaced in Brazil’s Acre and Goiás states, shedding light on a possible introduction route into Brazil, particularly from its border with Peru (Amorim et al. 2023). Our phylogenetic analysis revealed close relations between our sequences and those from the Tabatinga province in Brazil, suggesting a potential introduction from Tabatinga and subsequent northward spread into Colombia. Tabatinga, situated in the tripartite border region between Brazil, Colombia, and Peru, adjacent to Colombia’s Amazonas department, has been identified as a critical area for the introduction of new viral variants, as observed in the case of SARS-CoV-2 transmission (Ballesteros et al. 2021). The epidemiological implications of the circulation of this genotype in Colombia and Latin America are uncertain. Therefore, the importance of continuing surveillance of this variant is highlighted, given that according to previous studies, the introduction of a new variant in a region could result in the genotype becoming the most dominant and therefore an increase in cases.

However, it is important to recognize certain limitations in our study, such as the limited number of sampled departments, constraints in spatiotemporal sampling efforts, and the absence of DENV genomes from vectors. These limitations necessitate a more thorough and comprehensive analysis of the transmission dynamics of circulating variants in the country. Several limitations may influence the interpretation of our findings. Spatiotemporal sampling biases could potentially distort phylogenetic and phylogeographic reconstructions. Additionally, the absence of comprehensive clinical data for the samples, particularly a lack of vector genomes, restricts further analysis. Despite these limitations, and their impact on the depth of our interpretations, we believe that this study offers a valuable initial exploration of the genomic landscape of DENV in Colombia. Future studies incorporating enhanced spatiotemporal sampling strategies, coupled with robust genomic surveillance including vector genomes, are warranted to achieve a more comprehensive understanding.

Nevertheless, our findings regarding genomic epidemiology in the departments of Chocó, Norte de Santander, Meta, and Córdoba provide insights into DENV transmission dynamics and shed light on potential evolutionary patterns in our country. Nevertheless, it is crucial to underscore the significance of ongoing studies to ascertain DENV evolution, not just within Colombia but also in other Latin American countries that are currently impacted by a massive outbreak of dengue (Epidemiological Alert 2023). Moreover, there is a pressing need for further research dedicated to unraveling the intricacies of DENV virulence.

Finally, our genomic epidemiology study of DENV in Colombia underscores the crucial role of genomic epidemiology, employing advanced sequencing techniques such as ONT, as an indispensable tool for early detection of circulating DENV variants. This approach allows real-time identification of evolving genotypes and offers valuable insights into designing effective strategies to prevent and control potential outbreaks associated with the introduction of these variants. By integrating comprehensive objectives, including the complete genome characterization of the DENV, identification of circulating genotypes, and lineage characterization with genetic diversity assessment, this tool emerges as a robust mechanism for gaining a preliminary understanding of DENV evolution. We advocate utilizing this genomic epidemiology approach as an early warning system, facilitating the timely identification of circulating DENV variants, and leveraging genomic analysis to determine potential variants responsible for future outbreaks. The acquisition of sequencing capacity, such as third-generation sequencing, during the health emergency of the COVID-19, further strengthens our ability to employ this tool effectively.

## Supplementary Material

veae068_Supp

## Data Availability

All data used in this work are collected from NCBI and the information is in Supplementary Table S2. Some sequences generated in this study were deposited in NCBI under the accession numbers OR619404–405.
