# Supplementary figures and images for: Cryptic transmission and novel introduction of Dengue 1 and 2 genotypes in Colombia
Source: Virus Evol. 2024 Aug 31;10(1):veae068. doi: 10.1093/ve/veae068 (PMC11429525; doi:10.1093/ve/veae068)

Tree scale: 0.01

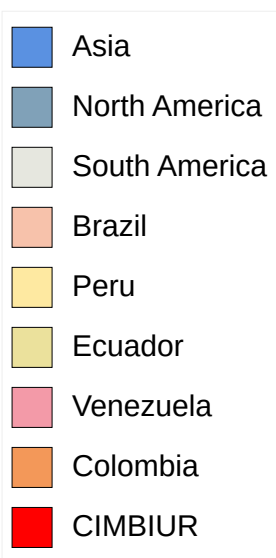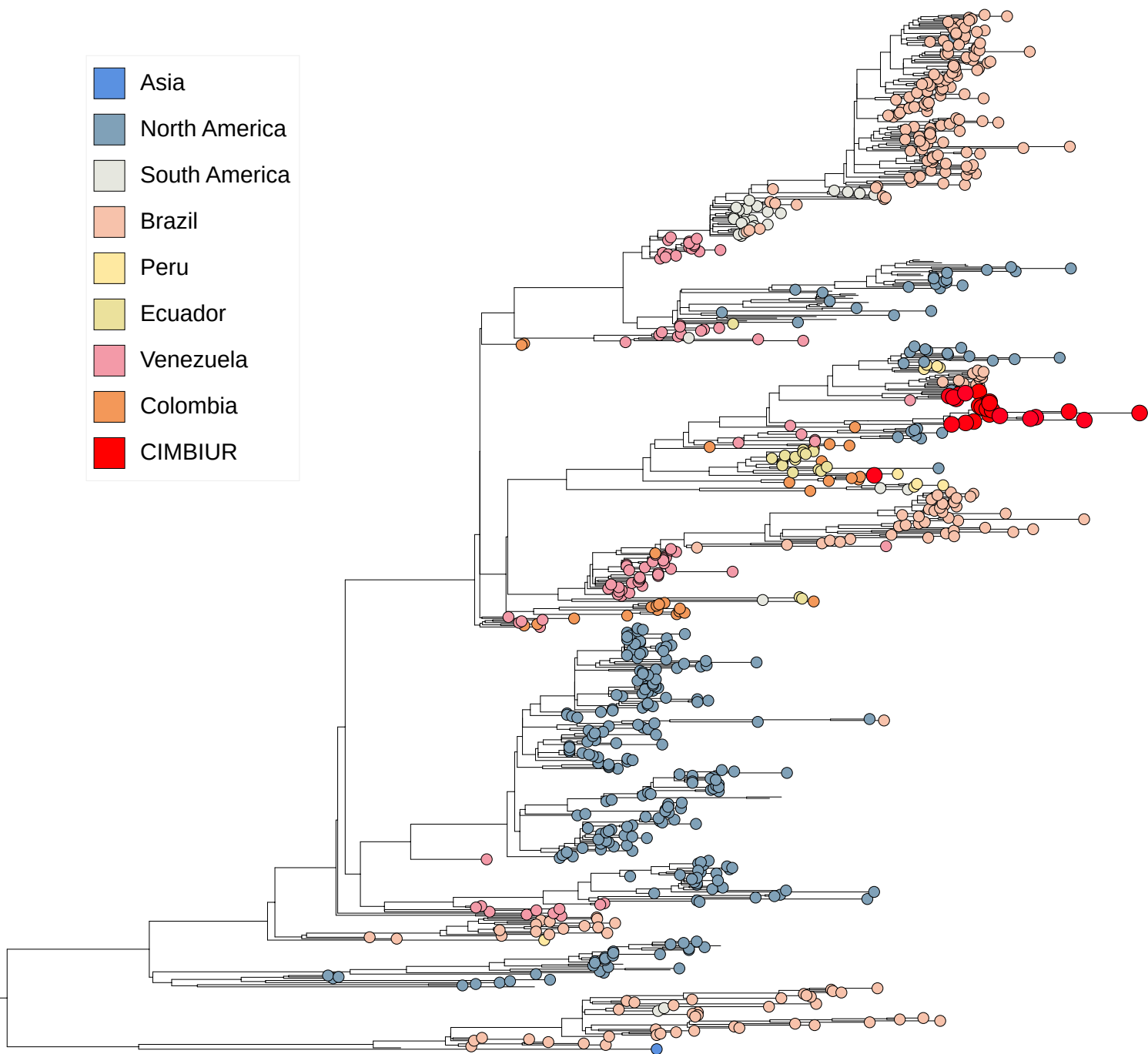

Supplement: veae068_Supp [file veae068_supp.zip › suppl_data/FigS1.pdf]

Tree scale: 0.01

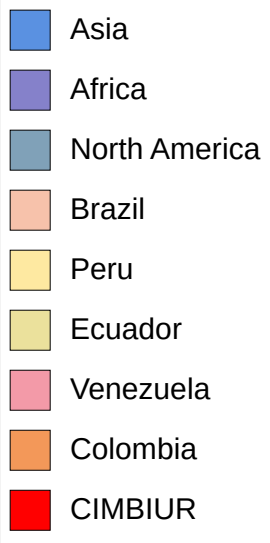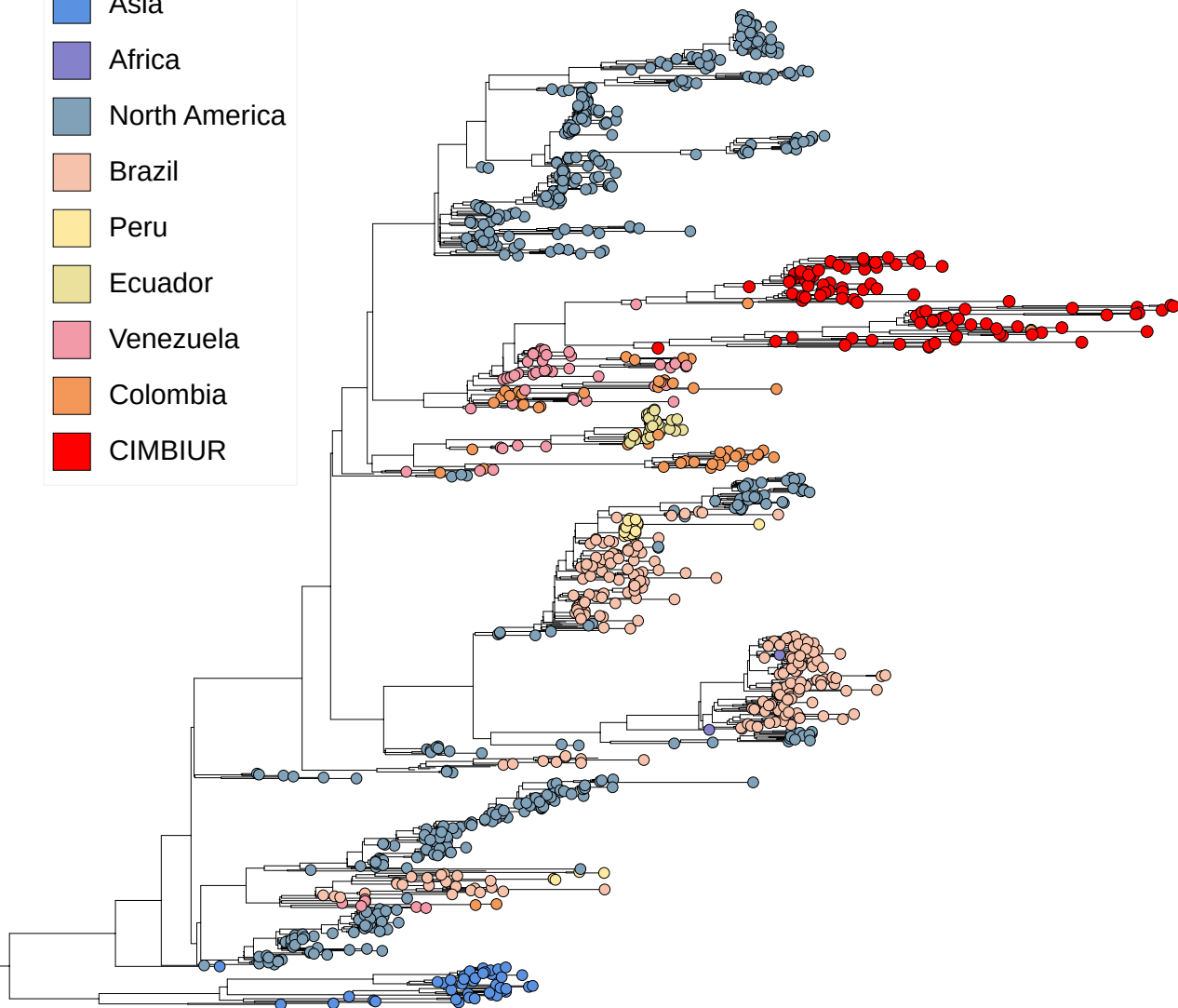

Supplement: veae068_Supp [file veae068_supp.zip › suppl_data/FigS2.pdf]

Tree scale: 0.007

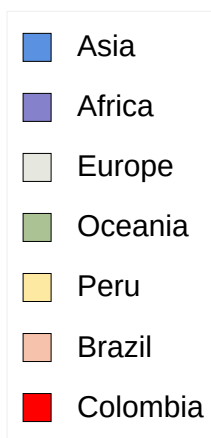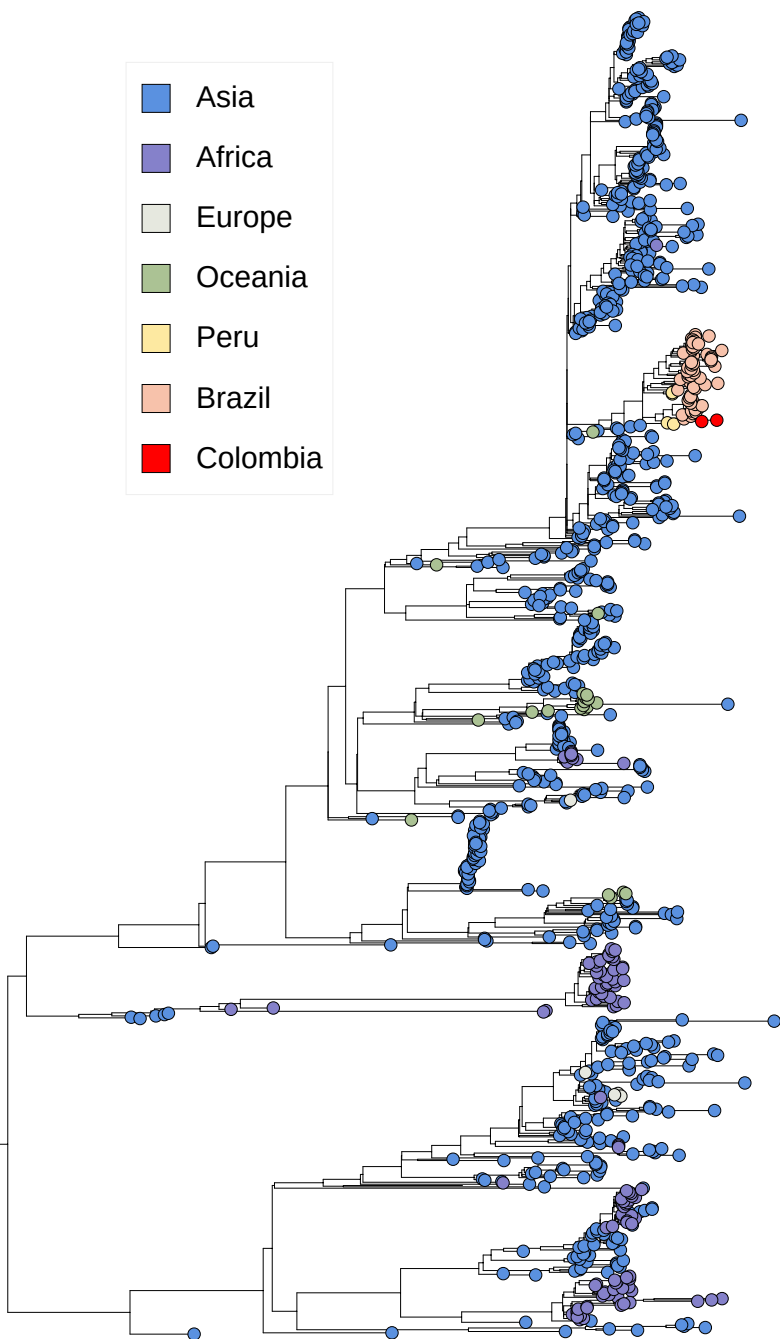

Supplement: veae068_Supp [file veae068_supp.zip › suppl_data/FigS3.pdf]
